# Modifications in the Consumption of Energy, Sugar, and Saturated Fat among the Mexican Adult Population: Simulation of the Effect When Replacing Processed Foods that Comply with a Front of Package Labeling System

**DOI:** 10.3390/nu10010101

**Published:** 2018-01-19

**Authors:** Rosario Mendoza, Lizbeth Tolentino-Mayo, Lucia Hernández-Barrera, Claudia Nieto, Eric A. Monterrubio-Flores, Simón Barquera

**Affiliations:** Centro de Investigación en Nutrición y Salud, Instituto Nacional de Salud Pública, Cuernavaca 62100, Mexico; rosario.mendoza@espm.insp.mx (R.M.); mltolentino@insp.mx (L.T.-M.); lhernan@insp.mx (L.H.-B.); claudia.nieto@insp.mx (C.N.); eric@insp.mx (E.A.M.-F.)

**Keywords:** front-of-package labeling, nutrition, processed foods, diet, obesity, Mexico

## Abstract

A Mexican Committee of Nutrition Experts (MCNE) from the National Institute of Public Health (INSP), free from conflict of interest, established food content standards to place the front-of-package (FOP) logo on foods that meet these nutrition criteria. The objectives were to simulate the effect on nutrient intake in the Mexican adult population (20–59 years old) after replacing commonly consumed processed foods with those that meet the FOP nutrition-labeling criteria. Twenty-four hour dietary recalls were collected from the 2012 Mexican National Health and Nutrition Survey (*n* = 2164 adults). A food database from the INSP was used. Weighted medians and 25–75 inter-quartile ranges (IQR) of energy and nutrient intake were calculated for all subjects by sociodemographic characteristics before and after replacing foods. Significant decreases were observed in energy (−5.4%), saturated fatty acids (−18.9%), trans-fatty acids (−20%), total sugar (−36.8%) and sodium (−10.7%) intake and a significant increase in fiber intake (+15.5%) after replacing foods, using the MCNE nutrition criteria. Replacing commonly consumed processed foods in the diet with foods that meet the FOP nutrition-labeling criteria set by the MCNE can lead to improvements in energy and nutrient intake in the Mexican adult population.

## 1. Introduction

Excessive body weight is one of the main public health problems in Mexico [1,2]. According to the 2016 National Survey of Health and Nutrition (ENSANUT, by its acronym in Spanish), the combined prevalence of overweight and obesity affects 72.5% of the Mexican population, representing 49.4 million people [3]. Obesity is recognized as a risk factor for non-communicable chronic diseases, such as diabetes mellitus and cardiovascular disease, which are the main causes of mortality in the country [4]. This growing obesity epidemic can be attributed to physical inactivity and significant changes in diet, such as the rise in consumption of processed foods, which has led to an increased intake of total fat, saturated fat, sugar and sodium, and a decrease in fiber intake among the population [2,5]. Although individuals have some responsibility for the quality of their diet, the environment can strongly influence decision-making for food selection and consumption [6,7].

Food labeling has been recognized as a tool with the potential to influence food choices and dietary habits of consumers [8,9]. Considering this, the Mexican government launched a clear front-of-package (FOP) nutrition-labeling system that provides useful information to guide people when selecting foods with a recommended nutrition content [10,11,12] and indirectly encourage industry to reformulate products.

In support of this strategy, the Mexican Committee of Nutrition Experts (MCNE) from the National Institute of Public Health (INSP, by its acronym in Spanish) established food content standards for energy and nutrients of concern, such as saturated fat, trans fat, added sugar, sodium and fiber. The MCNE proposed placing an FOP logo on foods that meet these nutrition criteria to distinguish them and classify them as “healthier” compared with the rest (see Table 1). The nutrition criteria were based on the dietary intake recommendations from the World Health Organization (WHO) [13] and the criteria previously established in the “Choices International” program, launched in The Netherlands in 2006 [14,15]. However, in 2014, the Ministry of Health in Mexico established the Guideline Daily Amounts (GDA) as mandatory, with voluntary use of a distinctive FOP logo on foods that meet the nutrition criteria of the Federal Commission for the Protection against Sanitary Risk (COFEPRIS, by its acronym in Spanish) [16] (see Table 2). However, these nutrition criteria were not based on the dietary intake recommendations from the WHO but were based on nutrition criteria set by the EU Pledge, an initiative of the food and beverage industry in the European Union [17]. The COFEPRIS nutrition criteria are not in line with international recommendations for a healthy intake and are far from the WHO criteria since they are based on the cut-off points established by the food and beverage industry.

The main goal of the present study was to assess which nutrition criteria could be a more effective strategy to regulate FOP nutrition-labeling in Mexico and to improve the nutrition intake of the Mexican population in the direction of the WHO recommendations, thus contributing to the prevention and control of overweight and obesity in the country. The specific objectives were (1) to simulate the effect on nutrient intake in the Mexican adult population (20–59 years old) after replacing normally consumed processed foods with those that meet FOP nutrition labeling criteria set by the MCNE and (2) to compare these results against the simulated effect of the nutrition criteria set by the COFEPRIS.

Simulation modelling was used to replace food products, as it has been recognized as an analytic methodology that uses data that estimate theoretical effects of a certain scenario on a set of outcomes [18]. Modelling is efficient when comparing hypothetical dietary scenarios; in this case, the intake of foods that comply with two different FOPL. Simulation methods can be used to predict dietary changes before they are implemented in populations. Therefore, this information could be translated and used in nutrition policies [19].

## 2. Materials and Methods

### 2.1. Design and Population

This cross-sectional study used the information from Mexican adults aged 20 to 59 years old who participated in the ENSANUT 2012 and whose dietary intake was measured by the 24-h recall (24HR) method. This survey is representative at the national level.

### 2.2. ENSANUT 2012

The ENSANUT is a complex design survey, in which data from demographic characteristics, health, nutrition and access to health services were collected from a nationally representative sample. The general datasets analyzed during the current study are available from the Mexican National Health and Nutrition Survey repository [20]. The detailed datasets analyzed during the current study are also available from the corresponding author on reasonable request. This survey had a complex probabilistic design with state representation by urban (population ≥ 2500 inhabitants) and rural (population < 2500 inhabitants) localities. The sampling frame was integrated with information from the Census of Population and Housing 2005, disaggregated by Geo-statistic Basic Areas and the list of newly emerging localities in the 2010 Census. Data collection of the ENSANUT 2012 was conducted between October 2011 and May 2012. Information on 50,528 households was obtained in each of the 32 states in the country, with a response rate of 87%. A detailed description of the sampling procedures and survey methodology has already been published elsewhere [21].

### 2.3. Dietary Information

Individual food consumption was obtained by the previously validated 24HR method [22,23,24]. The dietetic information was collected by standardized staff on a sub sample of 10,886 subjects (about 11% of the surveyed population of ENSANUT 2012); only those between the ages of 20 and 59 years were included in this analysis (*n* = 2281). This method consists of recording, through an interview, all food consumed by the individual the preceding day to estimate the average consumption of the population. An (iterative) multiple step or five-step method was used in order to capture more accurately the interviewee food intake and avoid underestimation. This method is an adapted version of the 24HR of the National Cancer Institute of the United States [22]. This method consists of five steps: (1) quick list of food consumed; (2) foods often forgotten; (3) time and occasion of consumption; (4) review and detail of ingested foods; and (5) final scan to help individuals completely and accurately remember all the foods eaten during the previous 24 h. Adults aged 20 to 59 years who had one completed 24HR were included in the analysis. Pregnant and/or lactating women (*n* = 98) and subjects with aberrant data such as a BMI under 10 kg/m^2^ or greater than 58 kg/m^2^ (*n* = 2) were excluded from the analysis. Aberrant dietary data were analyzed on a case-by-case basis and corrected when possible. Cases of individual consumption below −3 standard deviations (SD) and 3 SD above the average energy consumption and those with aberrant or missing data were excluded from analysis (*n* = 117, 0.78%) [25,26]. The final test sample consisted of 2164 individuals.

### 2.4. INSP Food Composition Database

An extensive and diverse food database assembled by researchers from the INSP (unpublished) [27] was used. The nutrition content of food was obtained from various sources such as the United States Department of Agriculture (USDA) [28], the Mexican Equivalent Food System [29], the nutrition tables of the National Institute of Medical Sciences and Nutrition Salvador Zubiran [30], standardized recipes and food labels. This database was used to determine the average intake of energy and macronutrients per capita per day, as well as to identify foods that meet FOP nutrition-labeling criteria from the MCNE and that are candidates to replace commonly consumed processed foods that do not meet these criteria. To calculate the added sugar amount in food, the USDA database of added sugar content in foods was used [31]. Finally, the nutrition content of new foods was obtained directly from product labels.

### 2.5. FOP Nutrition-Labeling Criteria

Processed foods reported in the 24HR were evaluated with MCNE nutrition criteria and COFEPRIS criteria. The description of each set of criteria is presented below.

#### 2.5.1. MCNE Nutrition Criteria

Foods were divided in 19 categories; cutoffs were set for the energy, saturated fat, trans fat, added sugar, sodium and fiber content per 100 g, 100 mL, or in % of total fat or energy of food. Cut-off points are different for each category (see Appendix A, Table A1).

#### 2.5.2. COFEPRIS Nutrition Criteria

Foods were divided into 26 categories, and limits were set for energy, saturated fat, total sugar and sodium content per 100 g or 100 mL, per serving or in % of total fat or energy per product. Cut-off points are different for each category. These criteria excluded sugar-based products, like chocolate products, jam, jelly, syrup, honey and soft drinks (see Appendix B, Table A2). Such food products are not able to carry the COFEPRIS logo because they are not considered healthy and are not supposed to comply with the cut-off points for nutrients of concern.

Both nutrition criteria groups were considered to classify foods into two groups: those that do not comply with the criteria and those that do comply and are candidates to replace the first ones. To measure the food replacement three scenarios were calculated and compared for both MCNE and COFEPRIS nutrition criteria:

Scenario 1: Represents the actual consumption. The median nutritional intake of the Mexican adult population based on data from a single 24HR per person from ENSANUT 2012.

Scenario 2: Simulates the food replacement. The median nutrient intake when replacing processed foods reported in a 24HR from ENSANUT 2012 that do not comply with the MCNE nutrition criteria, with similar foods that do comply with the criteria. When it was not possible to find a replacement, foods were not replaced. This allowed the maximum potential change in intake, adhering as much as possible to the consumption of foods reported.

Scenario 3: Simulates the food replacement with similar foods that did comply with criteria applying a correction factor with energy. Since foods that comply with criteria had less energy, the replacement could lead to a decrease in energy intake. For such a reason, the median intake of nutrients from Scenario 2 was corrected by the difference in energy density between the original intake and replacement, applying a correction factor. Therefore, when a food (for example, cereal bars, All Bran or Special K, 446.86 kcal/100 g) was replaced by a food with a lower energy density (in this case the Alpen light cereal bars, 290.5 kcal/100 g), a multiplication factor was applied (here 446.86/290.5 = 1.54) so that the total amount of energy consumed was the same as the amount of energy supplied by the food that had been replaced. This procedure was done for each product with the exception of sugar-sweetened beverages, bakery products and dairy, since correction would have resulted in unrealistic and very high amounts of consumption. Subsequently, the last two steps were repeated, but only replacing certain food groups or categories from the diet to see if significant differences were found in the intake of energy and nutrients after replacement using the MCNE nutrition criteria.

### 2.6. Statistical Analysis

Due to the skewed distribution, the energy and nutrient intake of the population were expressed in medians and weighted interquartile ranges (p25–p75). To evaluate statistically significant differences between the scenarios, energy and nutrient intake were log-transformed, and means were compared using linear regression models. A *p* value less than 0.05 was established to consider statistically significant differences. Statistical analyses were performed using Stata version 12.1, and the SVY module was used to adjust the sampling design of ENSANUT 2012 (College Station, TX, USA) [32].

### 2.7. Ethical Considerations

All participants from ENSANUT 2012 signed a pre-interview informed consent. This study protocol was reviewed and approved by the Ethics, Research and Biosafety Committee from the National Institute of Public Health.

## 3. Results

A total of 2164 adults aged 20–59 years were included in the analysis. The characteristics of the study sample are presented in Table 1. From the 695 foods and beverages that were identified in the 24HR, 354 items were classified as processed, excluding alcoholic beverages and dietary supplements. From the processed foods, 75.7% (*n* = 268) were classified as not meeting the MCNE nutrition criteria, and from these, only 44% (*n* = 118) could be replaced by a food that met the criteria; only those were used to calculate the nutrient intakes of Scenarios 2 and 3. From the total processed foods, only 45.6% (*n* = 167) were classified as not meeting the COFEPRIS nutrition criteria, and from these, 32.3% (*n* = 54) could be replaced by a food that met these nutrition criteria.

Median intakes of energy and nutrients before and after the replacement of food for both nutrition criteria are presented in Table 2. At the national level, energy, saturated fat, trans fat, total sugar, sodium and fiber intake showed significant changes (*p* < 0.05) when commonly consumed foods were replaced via simulation with those that met the MCNE criteria. When data were corrected for energy intake, the difference in nutrition intake was still evident, but was less for energy, saturated fat, trans fat, total sugar and sodium intake, and greater for fiber intake. When replacing foods using COFEPRIS criteria, only significant decreases were observed for trans fat and sodium intake (*p* < 0.05). When the simulated nutrition intake of both criteria was compared, a lower intake of energy, saturated fat and sugar, as well as a higher intake of fiber, was observed for the MCNE criteria, compared to the COFEPRIS criteria. No significant difference in trans fat and sodium intake between the two criteria was observed.

**Figure 1:**
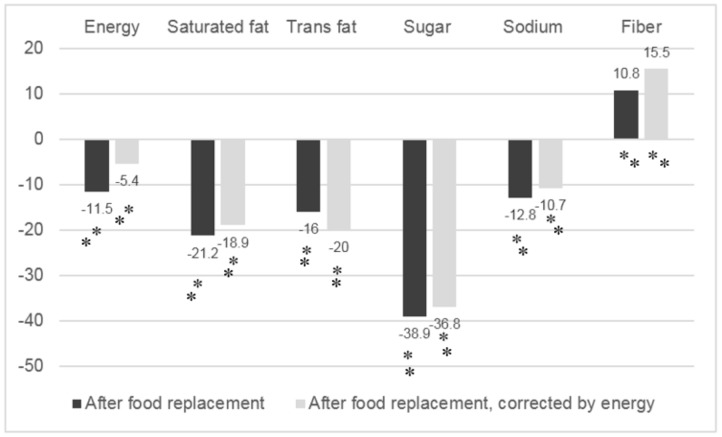
Median change of nutrient intake at national level after simulation of food replacement in the diet of the Mexican adult population (%) with FOP nutritional labeling criteria of the Mexican Committee of Nutrition Experts; * *p* > 0.05.

Figure 1 shows the percentage change in the median intake of energy, saturated fat, trans fat, sodium, total sugars and fiber, if the Mexican population were to replace all unhealthy processed foods in their diet with those that meet MCNE criteria (with and without adjustment of energy using a multiplication factor). Similar to the description of Scenario 3 when corrected by energy, the median energy intake was significantly reduced by 5.4%. In addition, significant reductions in the median intake of saturated fat (−18.9%), trans fat (−20%), sugar (−36.8%) and sodium (−10.7%) were observed, and median fiber intake increased by (15.5%). The greatest reduction was observed in the total sugar intake, both nationally and by demographic characteristics.

After testing the replacement of some food categories separately, only the replacement of sugar-sweetened beverages, using the MCNE criteria, produced a significant reduction in the sugar intake of the Mexican adult population (−28.3%). No significant differences in the rest of the nutrient and energy intakes were observed (data not shown).

## 4. Discussion

The FOP nutrition-labeling regulation is a strategy that may facilitate the adoption of healthy eating, by promoting better consumer decisions regarding the processed foods they consume, and thus helping to improve the nutrition and health status of the population [7,8]. This is the first study in Mexico that evaluates, through simulation, the potential impact of the replacement of commonly consumed foods in the diet, with products that meet FOP nutrition-labeling criteria, on the nutrition intake of the population. Some of the assumptions if consumers were to comply with the FOP nutrition-labeling criteria will be a significant decrease in the intake of dangerous nutrients such as, saturated fat, trans fat and sugar; furthermore, the largest and significant decrease will be that of sugar.

Based on the 24HR method, this analysis showed that if the Mexican adult population aged 20 to 59 years replaced processed foods commonly consumed in the diet with those that meet the MCEN nutrition criteria without changing other aspects of the diet, we would observe a significant decrease in the intake of energy, saturated fat, trans fat and total sugar. The excessive consumption of these critical nutrients for public health has been associated with a high prevalence of obesity and chronic non-communicable diseases such as diabetes mellitus, hypertension and dyslipidemias [33]. In contrast, we would observe a significant increase in fiber intake, of which adequate intake is associated with chronic disease reduction [34]. The subgroups that benefited the most from the replacement of food were men, adults 20–39 years old, those from urban locality, and the inhabitants of the northern region, possibly because of a greater consumption of processed foods than the rest. In contrast, those that least benefited from the replacement of food were those classified as underweight and normal weight, and those with low socioeconomic status; subgroups that may have had a lower consumption of processed foods compared to the rest.

Importantly, by using the MCNE criteria, the largest decrease was observed in sugar intake. This is because most of the replacement was made in sugar-sweetened beverages and the Mexican population, as has been observed and reported in previous studies, has a high consumption of these products [35]. When replacing only sugar-sweetened beverages for those with a very low sugar content, using the MCNE criteria, a significant decrease in the total sugar intake by the population was observed. This shows that by only replacing this group of beverages, a significant reduction in sugar intake could be observed, as well as a positive impact on the population health, since its excessive consumption has been associated with obesity and diabetes [35,36,37,38].

The results of this study, using the MCNE nutrition criteria, are consistent with those observed in similar studies, which performed a simulation of the replacement of processed foods by those that meet the criteria of the program “Choices International” (criteria on which the MCNE was based to establish their own). In 2009, Roodenburg et al., tested the potential impact of the replacement of processed foods in the diet with those that met the criteria of the “Choices International” program by modeling the nutrition intake of the Dutch adult population, observing substantial reductions in the intake of energy, saturated fat, trans fat, sugar and sodium, as well as an increase in fiber intake [39]. However, in this study, a greater reduction was observed in the intake of trans fat, compared with other nutrients, possibly because of a higher content in foods available on the Dutch market or food consumption patterns in the country. Later in 2011, a simulation of the replacement of processed foods in Greece, Spain, United States, Israel, China and South Africa was conducted. In this evaluation, three typical menus of each country based on population nutrient intake were assessed using the Choices program criteria. Three menus were then developed for each country replacing processed foods that did not meet the Choices criteria with those that did. This methodology showed that replacement may have a positive impact on reducing excess intake of nutrients with upper limits and increasing fiber intake among the adult population in the countries studied [40].

When simulation of replacing foods in the diet with those that meet the COFEPRIS nutrition criteria was performed, no significant decreases in the intake of energy, saturated fat and sugar were found, and no significant increase in fiber intake was found; only decreases in the intake of trans fats and sodium were observed. In addition, when decreases in the intake of energy, saturated fat, sugar and increase in fiber intake between MCNE and COFEPRIS criteria were compared, significant differences were found, finding a more favorable effect when using MCNE criteria. COFEPRIS nutrition criteria are based on those established by the food industry itself (which are not in accordance with the WHO dietary intake recommendations), being more permissible and less stringent than those of MCNE. Because of this, a greater number of products can be classified with a suitable nutrition profile, as observed in this analysis. With the MCNE criteria 25% of processed foods were classified as having an adequate nutrition content, while up to 56% of processed foods were classified in this category when COFEPRIS criteria were utilized. The latter causes consumers to perceive these products with a better nutrition quality and make unhealthy decisions regarding food choices. This would cause, as demonstrated in this study, negative positive impact on population nutrient intake and does not contribute to preventing and reversing obesity and non-communicable diseases in the country. Therefore, the COFEPRIS nutrition criteria are not considered an effective strategy for regulating FOP nutrition-labeling in Mexico.

Another important finding in this study was that a high proportion of processed foods consumed by Mexican adults (approx. 75%) does not meet the MCNE nutrition criteria, and less than half of these (44%) could be replaced with a healthier product that meets these criteria. For this reason, it is important not only to establish a more severe FOP nutrition-labeling regulation, but also to encourage the reformulation of processed foods by the industry. Some studies have found a successful reduction in nutrients of concern like sugar, saturated fat and sodium with stringent FOP labeling targets; also, studies showed that a logo perceived as credible and recognized helps in the choice of healthier foods [41,42,43]. At the same time, the population must be oriented towards a greater consumption of natural, traditional or low-level processing foods.

### 4.1. Strengths of the Study

Other studies have proved that modelling serves the purpose of accurately estimating the intake of nutrients when processed foods are replaced with healthier options [44,45,46]. These types of studies are often used to predict dietary changes before they are implemented in populations. Therefore, this information could be translated into nutrition policies and could potentially help policy makers to make better policies that will likely improve the health of the population and decrease diet-related non-communicable diseases [19].

### 4.2. Limitations of the Study

A limitation of the study is that we are not certain that people will replace foods with others that do comply with the criteria. This limitation is likely to happen in real life as people base their food choices and intakes not only on the FOP labeling but also on their activity and the whole environment [47].

Another limitation is that the dietary intake of the Mexican adult population in ENSANUT 2012 might be underestimated due to implications of the 24HR method [22], since it is difficult for a person to remember all foods and exact amounts consumed or the ingredients used in more complex food preparations. In addition, this questionnaire does not provide the variety or the exact amount of the usual food intake. This would result in a bias measurement of actual consumption. Furthermore, the database of the nutrient content of food may be inadequate because it uses different secondary sources that can provide, for some foods, a different nutrition content from the specific food consumed by the individual.

## 5. Conclusions

Based on simulations using data from the ENSANUT 2012, improvements can be observed in the energy and nutrient intake if Mexican adults replaced unhealthy processed foods commonly consumed in their diet with those that meet the MCNE nutrition criteria for FOP labeling. These results demonstrate the potential impact that could occur if the actual COFEPRIS nutrition criteria were adjusted towards the WHO recommendations, with emphasis on the category of sugar-sweetened beverages. This strategy, along with the establishment of clear food labeling, could be effective to regulate the FOP nutrition-labeling in Mexico, encouraging healthy decision-making regarding the purchase and consumption of foods, and thus improving the nutrient intake of the population.

## Figures and Tables

**Table 1:** Socio-demographic characteristics of participants. National Survey of Health and Nutrition (ENSANUT) 2012, Mexico.

	*n*	% ^+^
Sex		
Women	1258	52.1
Men	906	47.9
Age (years)		
20–39	1192	54.6
40–59	972	45.4
BMI *		
Underweight	22	0.7
Normal weight	564	28.2
Overweight	868	40.4
Obesity	710	30.7
Region		
North	544	19.8
South	769	31.1
Center and Mexico’s City	851	49
Locality		
Rural	756	25.3
Urban	1408	74.7
Socioeconomic level		
Low	776	29.4
Middle	703	30.4
High	685	40.2
Total	2164	100

^+^ Data adjusted by survey design; * BMI: Body Mass Index.

**Table 2:** Energy and nutrient intake before and after the simulation of food replacement in the diet of the Mexican adult population, with MCNE criteria and COFEPRIS criteria Mexico, ENSANUT 2012.

	With MCNE Criteria	With COFEPRIS Criteria
	National	National
	Median (p25–p75) ^+^	Median (p25–p75) ^+^
Energy (kcal)		
Scenario 1 Before replacement	1905 (1383–2477) ^a^	1905 (1383–2477) ^a^
Scenario 2 After replacement	1685 (1262–2236) ^b,^*	1821 (1330–2407) ^a,^*
Scenario 3 Corrected by energy	1802 (1276–2407) ^b,^*	1884 (1375–2465) ^a,^*
Saturated fat (g)		
Scenario 1 Before replacement	22 (13–33) ^a^	22 (13–33) ^a^
Scenario 2 After replacement	17 (10–26) ^b,^*	19 (12–30) ^a,^*
Scenario 3 Corrected by energy	18 (10–29) ^b,^*	21 (12–31) ^a,^*
Trans fat (g)		
Scenario 1 Before replacement	0.25 (0.05–0.59) ^a^	0.25 (0.05–0.59) ^a^
Scenario 2 After replacement	0.21 (0.04–0.49) ^a^	0.19 (0.03–0.46) ^b^
Scenario 3 Corrected by energy	0.2 (0.04–0.49) ^b^	0.2 (0.03–0.46) ^b^
Total Sugar (g)		
Scenario 1 Before replacement	86 (50–128) ^a^	86 (50–128) ^a^
Scenario 2 After replacement	52 (29–86) ^b,^*	84 (49–123) ^a,^*
Scenario 3 Corrected by energy	54 (31–86) ^b,^*	85 (49–126) ^a,^*
Sodium (mg)		
Scenario 1 Before replacement	2257 (1454–3384) ^a^	2257 (1454–3384) ^a^
Scenario 2 After replacement	1968 (1288–3084) ^b^	2006 (1274–3082) ^b^
Scenario 3 Corrected by energy	2014 (1311–3177) ^b^	2039 (1287–3156) ^b^
Fiber (g)		
Scenario 1 Before replacement	21 (14–32) ^a^	21 (14–32) ^a^
Scenario 2 After replacement	24 (16–35) ^b,^*	22 (15–32) ^a,^*
Scenario 3 Corrected by energy	25 (16–36) ^b,^*	23 (15–33) ^a,^*

MCNE: Mexican Committee of Nutrition Experts from the National Institute of Public Health; COFEPRIS: Federal Commission for Protection against Sanitary Risks; ^+^ Medians and percentiles. Scenario 1: Measured intake from ENSANUT 2012, before food replacement; Scenario 2: Measured intake after the replacement of commonly consumed processed food by those that meet the nutritional criteria of the MCNE; Scenario 3: Same measured intake as Scenario 2, adjusted by energy. ^a,b^ Different superscripts represent statistically significant differences against Scenario 1 (*p* < 0.05); * Statistically significant differences between MCNE nutritional criteria and COFEPRIS criteria (*p* < 0.05).

## Appendix A

**Table A1:** Front-of-package (FOP) nutrition labeling criteria of the Mexican Committee of Nutrition Experts (2010).

Food Group	Definition	Nutritional Criteria	
Processed food and vegetables	All types of processed fruit & vegetables, with the exception of fruit juices and frozen fruit & vegetables without further processing.	Saturated fat	≤1.1 g/100 g
		Trans fat	≤0.1 g/100 g
		Sodium Added sugar Fiber	≤100 mg/100 g
			Not added
			≥0.65 g/100 g
Pasta and potatoes	Includes all kinds of tubers, pastas and similar products used for main dishes.	Saturated fat	≤1.1 g/100 g
		Trans fat	≤0.1 g/100 g
		Sodium Added sugar Fiber	≤100 mg/100 g
			Not added
			≥4 g/100 g
Beans and other legumes	All kinds of beans and legumes: cooked, processed, fresh or dried.	Saturated fat	≤1.1 g/100 g
		Trans fat	≤0.1 g/100 g
		Sodium	≤250 mg/100 g
		Added sugar	Not added
		Fiber	≥3.5 g/100 g
Corn and tortilla products	Tortilla and corn products	Saturated fat	≤1.1 g/100 g
		Trans fat	≤0.1 g/100 g
		Sodium	≤100 mg/100 g
		Added sugar	Not added
		Fiber	≥3.5 g/100 g
Rice and other grains	All kinds of grain products such as rice, wheat oatmeal, amaranto, barely, products used for main dishes.	Saturated fat	≤1.1 g/100 g
		Trans fat	≤0.1 g/100 g
		Sodium	≤100 mg/100 g
		Added sugar	Not added
		Fiber	≥3.5 g/100 g
Salted bread	All kinds of bread or substitutes for bread with the exception of breakfast cereals.	Saturated fat	≤1.1 g/100 g
		Trans fat	≤0.1 g/100 g
		Sodium	≤500 mg/100 g
		Added sugar	≤13% energy
		Fiber	≥4 g/100 g
Sweet bread, pastry and cookies	All kinds of sweet baked goods, including industrialized and packaged rolls and pastries, and cereal bars.	Saturated fat	≤1.1 g/100 g
		Trans fat	≤0.1 g/100 g
		Sodium	≤300 mg/100 g
		Added sugar	≤13% energy
		Fiber	≥4 g/100 g
Breakfast cereal products	All kinds of breakfast cereal products.	Saturated fat	≤13% energy
		Trans fat	≤0.1 g/100 g
		Sodium	≤500 mg/100 g
		Added sugar	≤20 g/100 g
		Fiber	≥5 g/100 g
Milk and dairy products	All kinds of milk and dairy products.	Saturated fat	≤1.9 g/100 g
		Trans fat	≤0.1 g/100 g
		Sodium	≤100 mg/100 g
		Added sugar	≤6 g /100 g
Cheese (products)	All kinds of cheese and cheese products. Natural cheeses (fresh or aged), processed cheeses (American cheese).	Saturated fat	≤15 g/100 g
		Trans fat	≤0.1 g/100 g
		Sodium	≤400 mg/100 g
		Added sugar	Not added
Processed Meat (fresh and cured sausages and meat substitutes)	All kinds of processed meats, including beef, chicken and (vegetable) meat substitutes.	Saturated fat	≤1.1 g/100 g
		Trans fat	≤0.1 g/100 g
		Sodium	≤900 mg/100 g
		Added sugar	Not added
Fish and shellfish (fresh or frozen)	All kinds of fresh fish and shellfish (including deep-frozen without further processing or preparation).	Saturated fat	≤1.1 g/100 g ó 30% total fat
		Trans fat	≤0.1 g/100 g
		Sodium	≤100 mg/100 g
		Added sugar	Not added
Oils, fats and fat containing spreads	All fats and oils used as spreads or to prepare food.	Saturated fat	≤30% total fat
		Trans fat	≤1.3% energy
		Sodium	≤350 mg/ 100 g
		Added sugar	Not added
Soups	All kinds of soups and broths.	Saturated fat	≤1.1 g/100 g
		Trans fat	≤0.1 g/100 g
		Sodium	≤300 mg/100 g
		Added sugar	≤2.5 g/100 g
		Energy	≤100 kcal/100 g
Main course dishes	All Ready-to-Cook Meals that are intended to be eaten as main dish during lunch or dinner.	Saturated fat	≤1.1 g/100 g ó 13 in %
		Trans fat	≤0.1 g/100 g ó 1.3 in %
		Sodium	≤300 mg/100 g
		Added sugar	≤2.5 g/100 g ó 13% energy
		Fiber	≥1.25 g/100 kcal
		Energy	≤150 kcal/ 100 g
Sauces (water based)	All sauces that constitute only a minor component of the meal (portion size < 35 g) without added emulsifying agent and with fat content < 10% *w*/*w*.	Saturated fat	≤1.1 g/100 g
		Trans fat	≤0.1 g/100 g
		Sodium	≤550 mg/100 g
		Added sugar	Not added
		Energy	≤100 kcal/100 g
Sauces (emulsions)	All sauces that constitute only a minor component of the meal (portion size < 35 g) to which an emulsifying agent is added or have a fat content ≥ 10% *w*/*w*.	Saturated fat	≤1.1 g/100 g ó 30% energy
		Trans fat	
		Sodium	≤0.1 g/100 g ó 1.3% energy
		Added sugar	
			≤550 mg/100 g
		Energy	≤2.5 g/100 g ó 13% energy
			≤350 kcal/100 g
Snacks (savory and sweet)	All snack products intended to be eaten as a small snack between meals or as a minor component of a meal.	Saturated fat	≤1.1 g/100 g
		Trans fat	≤0.1 g/100 g
		Sodium	≤131 mg/100 g
		Added sugar	≤20 g/100 g
		Energy	≤300 kcal/100 g
Liquid foods	Products that are normally consumed form a cup, mug or glass (including products packed in portions in packages, bottles, etc.); includes beverages with fruit juices, with the exception of dairy products.	Saturated fat	≤1.1 g/100 mL
		Trans fat	≤0.1 g/100 mL
		Sodium	≤20 mg/100 mL
		Added sugar	Not added
		Energy	≤10 kcal/100 mL

## Appendix B

**Table A2:** FOP nutritional labeling criteria of the Commission for the Protection against Sanitary Risk (COFEPRIS 2014).

Food Group	Definition	Nutritional Criteria	
Fruits and vegetables, legumes, nuts, seeds and tubers (except snacks)	All types of fruits and vegetables, legumes, tubers, solid soy products (frozen, tined, dehydrated).	Saturated fat	≤1.5 g/100 g
		Sodium	≤300 mg/100 g
	Serving defined by COFEPRIS: 110 g	Total sugar	≤15 g/100 g
		Energy	≤170 kcal/serving
Fruit/vegetable based condiment	Fruit/vegetable-based condiments: >50 g fruit and/or vegetable per 100 g of finished products that constitute a minor component of the meal.	Saturated fat	≤1.5 g/100 g
		Sodium	≤750 mg/100 g
		Total sugar	≤25 g/100 g
	Serving defined by COFEPRIS: 20 g	Energy	≤85 kcal/serving
Soy liquid foods	Soy-made liquid foods with or without juice	Saturated fat	≤0.5 g/100 mL
		Sodium	≤110 mg/100 mL
		Total sugar	≤15 g/100 mL
	Serving defined by COFEPRIS: 200 mL		
		Energy	≤140 kcal/serving
Tortillas and corn products	All kinds of tortillas and corn products.	Saturated fat	≤5 g/100 g
		Sodium	≤670 mg/100 g
	Serving defined by COFEPRIS: 30 g	Added sugar	≤4 g/100 g
		Energy	≤300 kcal/serving
Cereals (other than made for breakfast)	Cereals other than made for breakfast (rice, pasta, bread, crackers).	Saturated fat	≤5 g/100 g
		Sodium	≤500 mg/100 g
	Serving defined by COFEPRIS: 50 g	Total sugar	≤5 g/100 g
		Energy	≤340 kcal/serving
Cakes	All kinds of cakes.	Saturated fat	≤10 g/100 g
		Sodium	≤450 mg/100 g
	Serving defined by COFEPRIS: 45 g	Total sugar	≤30 g/100 g
		Energy	≤190 kcal/serving
Sweet bread	All kinds of sweet bread.	Saturated fat	≤10 g/100 g
		Sodium	≤450 mg/100 g
	Serving defined by COFEPRIS: 50 g	Total sugar	≤30 g/100 g
		Energy	≤190 kcal/serving
Cookies	All kinds of cookies.	Saturated fat	≤10 g/100 g
		Sodium	≤450 mg/100 g
	Serving defined by COFEPRIS: 30 g	Total sugar	≤30 g/100 g
		Energy	≤160 kcal/serving
Cereal bars	All kinds of cereal bars.	Saturated fat	≤10 g/100 g
		Sodium	≤450 mg/100 g
	Serving defined by COFEPRIS: 30 g	Total sugar	≤35 g/100 g
		Energy	≤160 kcal/serving
Breakfast cereals	All kinds of breakfast cereals	Saturated fat	≤5 g/100 g
		Sodium	≤500 mg/100 g
	Serving defined by COFEPRIS: 30 g	Total sugar	≤30 g/100 g
		Energy	≤210 kcal/serving
Milk products	Dairy products other than cheeses.	Saturated fat	≤2.6 g/100 g
	Serving defined by COFEPRIS:	Sodium	≤300 mg/100 g
	-Liquids: 240 mL	Total sugar	≤13.5 g/100 g
	-Drinking yogurt: 200 mL	Energy	≤170 kcal/serving
	-Solid yogurt: 100 g		
Fresh cheese	Fresh cheese.	Saturated fat	≤10 g/100 g
		Sodium	≤800 mg/100 g
	Serving defined by COFEPRIS: 30 g	Total sugar	≤8 g/100 g
		Energy	≤70 kcal/serving
Mature cheese	Mature cheese.	Saturated fat	≤15 g/100 g
	Serving defined by COFEPRIS: 30 g	Sodium	≤900 mg/100 g
		Total sugar	≤5 g/100 g
		Energy	≤85 kcal/serving
Meat based products	Meat-based products, all kind of processed meat/poultry.	Saturated fat	≤6 g/100 g
		Sodium	≤800 mg/100 g
	Serving defined by COFEPRIS: 45 g	Total sugar	≤5 g/100 g
		Energy	≤170 kcal/serving
Fishery products	Fish and shellfish products.	Saturated fat	≤33% total fat
	Serving defined by COFEPRIS:	Sodium	≤450 mg/100 g
	-Fish: 50 g	Total sugar	≤5 g/100 g
	-Shellfish: 100 g		
		Energy	≤170 kcal/serving
Vegetable and animal fats and oils	All vegetable and animal fats and oils.	Poliunsaturated fat	≥ 25% total fat
			≤33% total fat
		Saturated fat	≤500 mg/100 g
		Sodium	
	Serving defined by COFEPRIS: 10 g	Total sugar	≤5 g/100 g
		Energy	≤85 kcal/serving
Soups	All kinds of soups.	Saturated fat	≤1.5 g/100 g
		Sodium	≤350 mg/100 g
	Serving defined by COFEPRIS: 200 mL	Total sugar	≤7.5 g/100 g
		Energy	≤170 kcal/serving
Dishes and sandwiches	Composed of dishes, main dishes and sandwiches.	Saturated fat	≤5 g/100 g
		Sodium	≤400 mg/100 g
	Serving defined by COFEPRIS: 200 g	Total sugar	≤7.5 g/100 g
		Energy	≤425 kcal/serving
Sauces for foods	Sauces for foods (>50 g of fruit and vegetables by 100 g of the total product).	Saturated fat	≤1.5 g/100 g
		Sodium	≤500 mg/100 g
		Total sugar	≤10 g/100 g
	Serving defined by COFEPRIS: 100 g	Energy	≤100 kcal/serving
Emulsions-based sauces	Emulsions-based sauces.	Saturated fat	≤33% total fat
		Sodium	≤750 mg/100 g
		Total sugar	≤5 g/100 g
	Serving defined by COFEPRIS: 15 g	Energy	≤85 kcal/serving
Salted snacks	Salted snacks.	Saturated fat	≤6 g/100 g
		Sodium	≤800 mg/100 g
	Serving defined by COFEPRIS: 45 g	Added sugar	≤5 g/100 g
		Energy	≤170 kcal/serving
Juices	All kinds of juices	Sodium	≤10 mg/100 mL
		Total sugar	≤13 g/100 mL
	Serving defined by COFEPRIS: 250 mL	Energy	≤130 kcal/serving
Nectars	All kinds of nectars	Sodium	≤28 mg/100 mL
		Total sugar	≤13 g/100 mL
	Serving defined by COFEPRIS: 200 mL	Energy	≤104 kcal/serving
Beverages with reduced energy content	Beverages with a reduced energy content.	Sodium	≤28 mg/100 mL
		Total sugar	≤5 g/100 mL
	Serving defined by COFEPRIS: 200 mL	Energy	≤40 kcal/serving
Ice cream and sorbets	All kinds of ice cream and sorbets	Saturated fat	≤5 g/100 g
		Sodium	≤120 mg/100 g
	Serving defined by COFEPRIS: 75 g	Total sugar	≤20 g/100 g
		Energy	≤110 kcal/serving
Jelly powders	Jelly powders to prepare jelly.	Saturated fat	≤5 g/100 g
		Sodium	≤120 mg/100 g
	Serving defined by COFEPRIS: 130 g	Total sugar	≤20 g/100 g
		Energy	≤110 kcal/serving
Foods excluded: sugar and sugar-based products; which include: chocolate products; jam or marmalade; sugar, honey or syrup; non-chocolate confectionary; soft drinks and other sugar products.			

## Appendix C

### Stratification for Analysis

The replacement effect was stratified nationally by sex (male or female), age group, BMI category, country region, locality and socioeconomic status only for substitution with MCNE nutrition criteria. Regarding the age, the sample was divided into two groups: 20 to 39 years old (young adult) and 40 to 59 years (middle adult). The WHO criteria were used to categorize the BMI into four groups: malnourished (<18.5 kg/m^2^), normal BMI (18.5 to 24.9 kg/m^2^), overweight (25.0–29.9 kg/m^2^) and obese (≥30.0 kg/m^2^) [48]. The locality was classified as rural if the population was <2500 inhabitants or urban if it had ≥2500 inhabitants. The country was divided into three regions: North, Central and Mexico City, and South. These three regions have common socio-economic and geographic characteristics and are grouped as follows: (A) North; (B) Centre; and (C) South. Finally, a socio-economic index was constructed using the Main Component Analysis with variables of the characteristics of living place, goods and services. Six variables (building materials of the floor and ceiling and possession of refrigerator, stove, television and computer) were selected. The first component that accounted for 41.1% of the total variability was selected as an index. Finally, socioeconomic status (SES) was classified into three categories using as cut-off points the percentiles 33 and 67 of the index (low, medium, high).

**Table A3:** Energy and nutrient intake at the national level by sex, age group, and BMI before and after food replacement in the diet of the Mexican adult population ^+^. Mexico, ENSANUT 2012.

	Sex		Group of Age		BMI			
	Women	Men	20–39	40–59	Underweight	Normal Weight	Overweight	Obesity
	Median (p25–p75)	Median (p25–p75)	Median (p25–p75)	Median (p25–p75)	Median (p25–p75)	Median (p25–p75)	Median (p25–p75)	Median (p25–p75)
Energy (kcal)								
1	1690	2134.4 (1631–2939.2) ^a^	1985.4 (1455.4–2624.4) ^a^	1787.1 (1313.4–2326.2) ^a^	2857.9 (2077.5–3160.6) ^a^	2013.8 (1468.6–2786.9) ^a^	1890.5 (1403.3–2424.6) ^a^	1860.5 (1320.5–2326.2) ^a^
	(1261.7–2153.7) ^a^							
2	1522.3	1916.9 (1417.7–2690.2) ^b^	1754.9 (1289.7–2355.3) ^b^	1628.8 (1222–2166.8) ^a^	2601.7 (1958.8–2853.1) ^a^	1853.8 (1317.1–2457.7) ^a^	1670.9 (1279.2–2201.1) ^b^	1637.7 (1196.6–2102.1) ^b^
	(1153.3–2004.3) ^b^							
3	1612.1	2017.7 (1393.6–2755.8) ^b^	1876.3 (1322.2–2511.1) ^b^	1722.3 (1199.4–2324) ^a^	3104.1 (1834.8–3406.2) ^a^	1924.7 (1351.9–2533.3) ^a^	1803.6 (1263.2–2398) ^a^	1716.7 (1197.3–2222.2) ^a^
	(1186.8–2084.5) ^a^							
Saturated Fat (g)								
1	19.7 (11.2–28.5) ^a^	24.5 (14.8–37.8) ^a^	23.1 (14.1–36.1) ^a^	20 (11–29.4) ^a^	49 (20.4–52) ^a^	23 (12.5–35.7) ^a^	21.6 (12.2–32.7) ^a^	20.6 (12.6–30) ^a^
2	15.2 (8.8–23.6) ^b^	19.8 (12.2–31) ^b^	18.1 (11.7–29.6) ^b^	15.8 (8.9–24.7) ^b^	21.8 (15.9–38.1) ^a^	18.1 (10.5–29.5) ^b^	17.1 (10.1–25.4) ^b^	16.8 (9–25.3) ^b^
3	15.7 (9.3–25.7) ^b^	20.5 (11.8–32) ^b^	19.3 (11–30.5) ^b^	16.2 (9.5–26.7) ^b^	28.3 (16–42.4) ^a^	19.9 (10.8–29.5) ^a^	17.5 (9.9–27.4) ^b^	15.9 (9.5–28.1) ^b^
Trans fat (g)								
1	0.24 (0.05–0.5) ^a^	0.26 (0.05–0.64) ^a^	0.26 (0.06–0.64) ^a^	0.23 (0.04–0.53) ^a^	0.35 (0.31–0.63) ^a^	0.27 (0.05–0.65) ^a^	0.24 (0.05–0.56) ^a^	0.26 (0.06–0.56) ^a^
2	0.2 (0.04–0.42) ^a^	0.22 (0.04–0.54) ^a^	0.22 (0.05–0.5) ^a^	0.19 (0.04–0.46) ^a^	0.33 (0.29–0.6) ^a^	0.23 (0.04–0.53) ^a^	0.19 (0.04–0.45) ^a^	0.19 (0.05–0.46) ^a^
3	0.2 (0.04–0.48) ^a^	0.2 (0.03–0.5) ^b^	0.19 (0.4–0.49) ^a^	0.21 (0.04–0.5) ^a^	0.62 (0.2–1.08) ^a^	0.22 (0.04–0.59) ^a^	0.18 (0.04–0.44) ^a^	0.18 (0.04–0.43) ^a^
Tot sugar (g)								
1	73.1 (43.8–117.7) ^a^	95.3 (58.2–138.6) ^a^	94.1 (53.6–136.9) ^a^	80.1 (43.7–115.8) ^a^	161.3(99.5–178.7) ^a^	86.1 (52.4–130.2) ^a^	82.9 (47.2–123.2) ^a^	88.1 (49–128.5) ^a^
2	51.1 (29.2–78.5) ^b^	53.1 (28.6–89.5) ^b^	54.3 (28.6–88.5) ^b^	49.5 (29.2–84.2) ^b^	111.8(74.6–135.7) ^a^	55.5 (29.8–89.5) ^b^	51 (28.4–84.5) ^b^	48.9 (28.5–80.8) ^b^
3	55.4 (31.8–83.9) ^b^	53.1 (30.3–89.5) ^b^	55.6 (28.9–90.8) ^b^	53.1 (32.7–82.8) ^b^	92.7 (63.2–111.6) ^a^	60 (33.3–90.6) ^b^	53.5 (30.8–85.5) ^b^	51.7 (28.1–81) ^b^
Sodium (mg)								
1	1967.9	2570.8 (1640.9–3866.8) ^a^	2313.7 (1507.2–3565.7) ^a^	2141.4 (1344.2–3212.4) ^a^	2737 (1550.3–5644.6) ^a^	2476.7 (1508–3766.1) ^a^	2141.4 (1377.6–3215.1) ^a^	2281.1 (1454.8–3220) ^a^
	(1271.7–2955.4) ^a^							
2	1792.3	2259.9 (1464.3–3460.7) ^a^	2061.9 (1370.6–3184.8) ^a^	1848.1 (1212.9–2984.7) ^a^	2329.1 (1102–5620.8) ^a^	2293.7 (1424.6–3455) ^a^	1868.4 (1286.9–2928.7) ^a^	1968.2 (1208.9–2942.1) ^a^
	(1170.8–2686.7) ^a^							
3	1889	2228.2 (1435.3–3627.7) ^a^	2114.3 (1321.8–3304) ^a^	1922 (1264.1–3062.3) ^a^	2348 (1221.1–3458.4) ^a^	2240.5 (1361.1–3542.3) ^a^	1938 (1253–3117.8) ^a^	1981.5 (1348.7–2970.9) ^a^
	(1170.4–2818.6) ^a^							
Fiber (g)								
1	20.1 (13.2–28.2) ^a^	23.4 (14.4–35.4) ^a^	20.3 (12.8–30.7) ^a^	22.1 (14–32.9) ^a^	17.5 (12.7–29) ^a^	21.4 (14.8–35) ^a^	21.9 (13.3–31.6) ^a^	19.8 (13–29.8) ^a^
2	22.2 (15–31.5) ^b^	26.3 (17–37.4) ^b^	22.9 (16.4–33.7) ^b^	24 (15.5–35.7) ^a^	29 (20.9–37.4) ^a^	24.5 (17.4–36.5) ^a^	23.7 (15.7–35.1) ^a^	22.7 (14.6–32.9) ^a^
3	23.3 (15.4–34) ^b^	26.4 (16.7–38.4) ^a^	24.9 (16.4–36.9) ^b^	24.5 (15.3–35.6) ^a^	24.4 (18.6–48.1) ^a^	25.5 (17.5–37) ^a^	24.8 (15.3–36.5) ^a^	23.3 (15.2–35.3) ^a^

1: Scenario 1, measured intake from ENSANUT 2012 before food replacement; 2: Scenario 2, measured intake after the replacement of commonly consumed processed food by those that meet the nutritional criteria of the Mexican Committee of Nutrition Experts; 3: Scenario 3, same measured intake as Scenario 2, adjusted by energy; ^a,b^ Different superscripts represent statistically significant differences with respect to Scenario 1 (*p* < 0.05); ^+^ Medians and weighted interquartile ranges.

## Appendix D

**Table A4:** Energy and nutrient intake by locality, country region and socio-economic level before and after food replacement in the diet of the Mexican adult population ^+^. Mexico, ENSANUT 2012.

	Locality		Region			Socio-Economic Level		
	Rural	Urban	North	South	Center and Mexico’s City	Low	Middle	High
	Median (p25–p75)	Median (p25–p75)	Median (p25–p75)	Median (p25–p75)	Median (p25–p75)	Median (p25–p75)	Median (p25–p75)	Median (p25–p75)
Energy (kcal)								
1	1846.3	1915.4	1906.1	1922.4	1869.1	1861	1928.1	1915.5
	(1320.8–2393.5) ^a^	(1403.3–2519.2) ^a^	(1428.4–2547.6) ^a^	(1391.5–2486.7) ^a^	(1335.2–2399.9) ^a^	(1348.7–2424.6) ^a^	(1403–2521.7) ^a^	(1381–2491.4) ^a^
2	1659.4	1692	1643.2	1770.9	1645.9	1719.2	1657.9	1689.2
					(1253.6–2203.4) ^b^			
	(1232.5–2166.8) ^a^	(1266.8–2276.7) ^b^	(1205.2–2210.5) ^b^	(1300–2340) ^a^		(1280.2–2194.8) ^a^	(1253.6–2276.6) ^b^	(1227.7–2281.5) ^b^
3	1745.6	1815.8	1762	1841.4 (1265.8–2476.9) ^a^	1790.6	1744.8	1836.2	1815.5
	(1233.4–2323.3) ^a^	(1291.4–2427.2) ^b^	(1199.7–2431.6) ^b^		(1297.9–2367.8) ^a^	(1204.3–2363.3) ^a^	(1322.2–2461.7) ^a^	(1296.6–2371.7) ^a^
Saturated fat (g)								
1	16.9 (9.3–26.9) ^a^	22.9 (13.9–35.3) ^a^	22.6 (13.1–34.7) ^a^	19.1 (10.7–30.3) ^a^	22.4 (13.4–33.7) ^a^	16.8 (9.2–27.3) ^a^	22.8 (13.3–33.3) ^a^	24 (14.8–36.3) ^a^
2	14 (7.8–22.1) ^b^	18.1 (11.3–28.1) ^b^	17.4 (9.6–26.9) ^b^	15.5 (9.3–24.2) ^b^	18.1 (11.2–28) ^b^	14.2 (7.5–22.5) ^b^	18.1 (10.5–26.2) ^b^	18.7 (12–29.4) ^b^
3	14.3 (8.1–22.7) ^b^	19.3 (10.8–30) ^b^	17.6 (10–28.8) ^b^	15.9 (9–26) ^b^	18.7 (10.5–30.7) ^b^	14.8 (8.1–24.5) ^a^	18.7 (10.1–28.5) ^b^	19.8 (12.3–30.4) ^b^
Trans fat (g)								
1	0.15 (0.02–0.43) ^a^	0.29 (0.07–0.62) ^a^	0.19 (0.04–0.53) ^a^	0.23 (0.04–0.5) ^a^	0.29 (0.07–0.63) ^a^	0.15 (0.03–0.41) ^a^	0.26 (0.06–0.54) ^a^	0.29 (0.09–0.65) ^a^
2	0.12 (0.02–0.39) ^a^	0.23 (0.05–0.53) ^a^	0.16 (0.04–0.48) ^a^	0.2 (0.03–0.42) ^a^	0.23 (0.05–0.53) ^a^	0.12 (0.02–0.39) ^a^	0.23 (0.04–0.46) ^a^	0.24 (0.06–0.59) ^a^
3	0.12 (0.02–0.36) ^a^	0.23 (0.05–0.54) ^a^	0.14 (0.03–0.53) ^a^	0.18 (0.03–0.42) ^a^	0.23 (0.05–0.51) ^a^	0.13 (0.03–0.42) ^a^	0.16 (0.03–0.45) ^a^	0.27 (0.06–0.55) ^a^
Total Sugar (g)								
1	67.1 (36–105.6) ^a^	92.8 (54.4–135.8) ^a^	91 (53.9–130.8) ^a^	76.5 (41.3–120.9) ^a^	88.7 (54.4–130.2) ^a^	69.3 (36.1–112.6) ^a^	86.9 (47–132.2) ^a^	94.1 (63–136.3) ^a^
2	46.5 (26.8–71.1) ^b^	54.3 (30.3–89.4) ^b^	45.2 (27.8–78.2) ^b^	50.3 (27.1–84.2) ^b^	55.3 (32.6–88.3) ^b^	43.6 (23.9–71.1) ^b^	48 (27.5–78.5) ^b^	60 (37.8–97.2) ^b^
3	47.2 (26.3–75.7) ^b^	57.7 (32–89.8) ^b^	50.4 (28.5–82.5) ^b^	51.6 (27.9–83.3) ^b^	59 (34.4–88.7) ^b^	44.2 (25.7–75.6) ^b^	52.8 (28.3–85.8) ^b^	65.8 (36.5–92.7) ^b^
Sodium (mg)								
1	1998.3	2306	2340.8	1992.6	2318.1	2010.4	2308.6	2340.8
	(1221.1–3231.9) ^a^	(1521.5–3422.9) ^a^	(1521.7–3660.3) ^a^	(1249.1–3121.4) ^a^	(1500.5–3422.9) ^a^	(1223.1–3236.6) ^a^	(1450.3–3509.8) ^a^	(1596.4–3422.9) ^a^
2	1789	2033.1	1988.1	1839.3	2121.7	1817.2	1963.8 (1288–3208.1) ^a^	2071.1
	(1107.4–2860.8) ^a^	(1360.5–3171.7) ^b^	(1293.3–3192.2) ^a^	(1136.4–2823.6) ^a^	(1392.5–3208.1) ^a^	(1107.4–2901.6) ^a^		(1423.7–3172.5) ^a^
3	1874	2052.9	2086.6	1824.1	2163.5	1922.5	2020.4	2142.9
					(1378.5–3421.4) ^a^			
	(1172.3–3042.3) ^a^	(1358.7–3235.7) ^a^	(1352.3–3189.6) ^a^	(1200.8–2874.4) ^a^		(1201.8–3041.8) ^a^	(1229.1–3161.7) ^a^	(1501.5–3381.7) ^a^
Fiber (g)								
1	25.1 (15.2–36.2) ^a^	20.3 (12.8–30.4) ^a^	17 (11.4–28) ^a^	24.1 (15.9–35.8) ^a^	21.2 (13.9–30.6) ^a^	24.4 (15.1–36.7) ^a^	21.2 (13.2–31.9) ^a^	19.7 (12.5–29.3) ^a^
2	27.5 (17.3–37.9) ^a^	22.8 (15.4–33.1) ^b^	21.3 (14–30.6) ^b^	26.6 (17.3–37.6) ^a^	23.1 (16.2–33.5) ^a^	26.4 (17.9–37.8) ^b^	22.6 (15.4–33.8) ^b^	21.9 (15.2–32.6) ^b^
3	27.1 (16.3–40) ^a^	23.9 (16–35.2) ^b^	23 (13.8–33.2) ^b^	27 (17–38.2) ^a^	23.9 (16–36.4) ^a^	27 (16.6–39.4) ^a^	24.2 (16.1–35.8) ^a^	23.4 (15.9–34.5) ^b^

1: Scenario 1, measured intake from ENSANUT 2012 before food replacement; 2: Scenario 2, measured intake after the replacement of commonly consumed processed food by those that meet the nutritional criteria of the Mexican Committee of Nutrition Experts; 3: Scenario 3, same measured intake as Scenario 2, adjusted by energy; ^a,b^ Different superscripts represent statistically significant differences with respect to Scenario 1 (*p* < 0.05); ^+^ Medians and weighted interquartile ranges.
